# Resveratrol treatment reveals a novel role for HMGB1 in regulation of the type 1 interferon response in dengue virus infection

**DOI:** 10.1038/srep42998

**Published:** 2017-02-20

**Authors:** Nurhafiza Zainal, Chih-Peng Chang, Yi-Lin Cheng, Yan-Wei Wu, Robert Anderson, Shu-Wen Wan, Chia-Ling Chen, Tzong-Shiann Ho, Sazaly AbuBakar, Yee-Shin Lin

**Affiliations:** 1 Institute of Basic Medical Sciences, College of Medicine, National Cheng Kung University, Tainan, Taiwan; 2 Department of Microbiology and Immunology, College of Medicine, National Cheng Kung University, Tainan, Taiwan; 3 Institute of Graduate Studies, University of Malaya, Kuala Lumpur, Malaysia; 4 Center of Infectious Disease and Signaling Research, National Cheng Kung University, Tainan, Taiwan; 5 Departments of Microbiology & Immunology and Pediatrics, and Canadian Center for Vaccinology, Dalhousie University, Halifax, Nova Scotia B3H 4R2, Canada; 6 Translational Research Center, Taipei Medical University, Taipei, Taiwan; 7 Department of Pediatrics, National Cheng Kung University Hospital, Tainan, Taiwan; 8 Graduate Institute of Clinical Medicine, College of Medicine, National Cheng Kung University, Tainan, Taiwan; 9 Tropical Infectious Diseases Research and Education Centre (TIDREC), University of Malaya, Kuala Lumpur, Malaysia; 10 Department of Medical Microbiology, Faculty of Medicine, University of Malaya, Kuala Lumpur, Malaysia

## Abstract

Dengue is one of the most significant mosquito-borne virus diseases worldwide, particularly in tropical and subtropical regions. This study sought to examine the antiviral activity of resveratrol (RESV), a phytoalexin secreted naturally by plants, against dengue virus (DENV) infection. Our data showed that RESV inhibits the translocation of high mobility group box 1 (HMGB1), a DNA binding protein that normally resides in the nucleus, into the cytoplasm and extracellular milieu. HMGB1 migrates out of the nucleus during DENV infection. This migration is inhibited by RESV treatment and is mediated by induction of Sirt1 which leads to the retention of HMGB1 in the nucleus and consequently helps in the increased production of interferon-stimulated genes (ISGs). Nuclear HMGB1 was found to bind to the promoter region of the ISG and positively regulated the expression of ISG. The enhanced transcription of ISGs by nuclear HMGB1 thus contributes to the antiviral activity of RESV against DENV. To the best of our knowledge, this is the first report to demonstrate that RESV antagonizes DENV replication and that nuclear HMGB1 plays a role in regulating ISG production.

Dengue, a systemic virus infection is transmitted between humans by *Aedes* mosquitoes. More than one-third of the world’s population is at risk of dengue with an estimated 390 million infections including 96 million clinically apparent cases annually^1^. Dengue virus (DENV) consists of 4 antigenically distinct serotypes, namely DENV-1, DENV-2, DENV-3 and DENV-4. Dengue manifestation involves symptoms ranging from mild dengue fever to more severe conditions such as dengue hemorrhagic fever (DHF) and dengue shock syndrome (DSS). The pathogenesis of dengue has been extensively investigated, although it is not completely understood. Improved insights into the pathogenesis of dengue are an important factor in designing safe and effective vaccines and antiviral drugs against the virus.

Innate immune responses are crucial for the host to restrict virus replication and limit infection. Host-pathogen recognition leads to the production of type-1 interferon (IFN), triggering a signaling cascade involving activation of JAK protein tyrosine kinases, STAT phosphorylation, and the stimulation of interferon-stimulated genes (ISGs). Type 1 IFN does not have the capability to inhibit virus replication directly, but instead activates hundreds of ISGs to induce a significant antiviral state^2^. ISGs both directly and indirectly inhibit viral infection by targeting various stages in the viral life cycle. Some ISGs such as Mx1/MxA disrupt early stages preceding genome replication, whereas ISG15 is involved in antiviral post-translational alteration via ISGylation^3^,^4^. Strategies designed to exploit or modulate specific ISG expression may be effective tools in the development of more effective antiviral agents.

Resveratrol (3, 5, 4′-trihydroxy-*trans*-stilbene; RESV), a widely known anti-inflammatory and antioxidant agent, is a phytoalexin synthesized by plants as one of the defense mechanisms against infection and stress. There are varied health benefits of RESV including protective activity against cardiac diseases, neurodegenerative and metabolic disorders, as well as chemopreventive features^5^. Previous studies showed that RESV inhibits replication of enterovirus 71, influenza A virus, respiratory syncytial virus, human immunodeficiency virus and Epstein-Barr virus^6^,^7^,^8^,^9^,^10^. However, the antiviral activity of RESV for DENV is still unknown. In order to investigate the potential antiviral properties of RESV for DENV, we are focusing on the effects of high mobility group box 1 (HMGB1), a protein which is found to be notably affected by RESV treatment^11^ and which is also involved in the pathogenesis of other viral infection^12^,^13^.

HMGB1 is a non-histone, DNA-binding protein that predominantly resides in the nucleus and is involved in regulation of transcription and DNA repair, as well as chromatin stability. During cell activation, infection, injury or death, HMGB1 translocates from the nucleus into the cytoplasm and eventually out of the cell^14^. The migration of HMGB1 out of the nucleus is triggered by acetylation of HMGB1 that leads to its release. Once released from the cell, HMGB1 binds to receptors such as toll-like receptor 2 (TLR2), TLR4 or receptor for advanced glycation end products (RAGE), which then leads to activation of multiple networks of inflammatory reactions^15^. Although HMGB1 has been found to have an impact on the replication of several viruses, the underlying mechanisms are poorly understood.

The level of HMGB1 has been reported to be significantly higher in the sera of dengue patients than in healthy controls^16^. A previous study showed the passive release of HMGB1 from DENV-induced necrosis of epithelial cells^17^. In contrast, another study reported release of HMGB1 from DENV-infected dendritic cells, under non-necrotic conditions^18^. During DENV infection, the translocation of HMGB1 was found to be facilitated by monocytic cell p300/CBP-associated factor (PCAF) acetylase complex that is activated by virus capsid protein^19^. The migration of HMGB1 from the nucleus to the cytoplasm is triggered by HMGB1 acetylation that leads to its release into the extracellular milieu. The released HMGB1 may bind to RAGE receptors on endothelial cells, which then contributes to the loss of vascular integrity^19^.

Treatment with RESV inhibits HMGB1 translocation out of the nucleus^20^, via induction of sirtuin 1 (Sirt1), a NAD^+^-dependent class III protein deacetylase^11^. RESV functions as the activator of Sirt1 and initiates the upregulation of the enzyme^21^. Sirt1, a mammalian ortholog of yeast silent information regulator 2, is conserved in most cells and is mainly present in the nucleus. Sirt1 is involved in various metabolic and pathophysiological processes, such as mitochondrial biogenesis, cellular senescence, energy metabolism, stress resistance, and inflammation^22^,^23^,^24^. Recent studies showed that upregulation and activation of Sirt1 inhibits LPS-induced HMGB1 release *in vitro* and *in vivo*^25^. Deacetylation of HMGB1, which leads to the retention of HMGB1 in the nucleus, is initiated by the interaction of Sirt1 protein with multiple lysine residues at NLS sites of HMGB1^26^. Furthermore, RESV treatment augments Sirt1 production in the nucleus and subsequently deacetylates HMGB1, which leads to nuclear HMGB1 accumulation^11^. This study aimed to examine the antiviral activity of RESV against DENV and determine its mechanism.

## Results

### RESV exerts a negative effect on DENV replication

**Figure 1:**
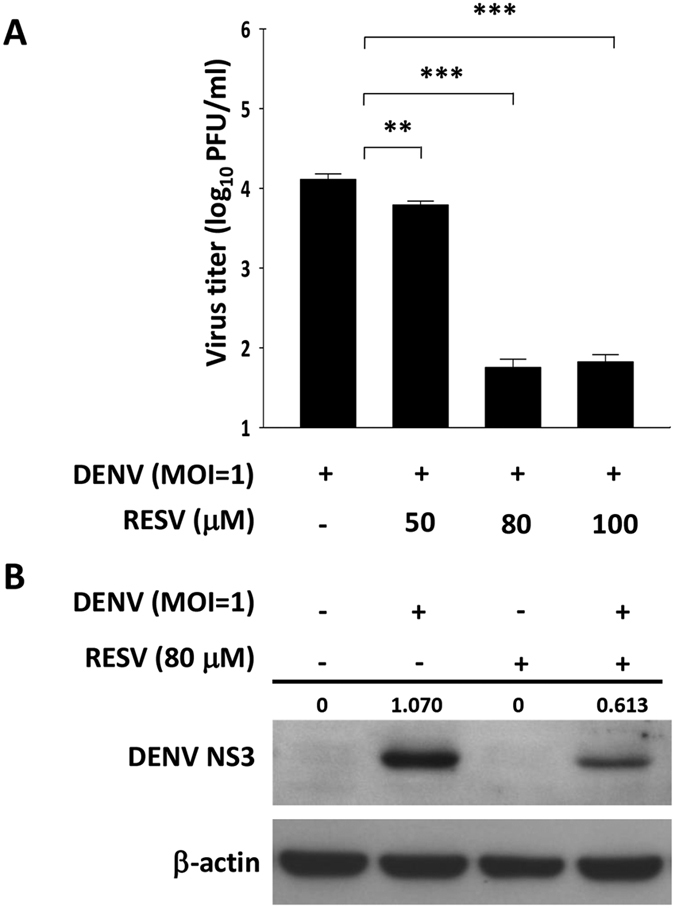
RESV treatment reduces virus replication in DENV-infected cells. (**A**) Huh7 cells were mock-treated or treated with 50, 80 or 100 μM of RESV after DENV infection at an MOI of 1 for 24 h. The cell supernatants were collected and analyzed for virus titers using plaque assay. Statistically significant differences between the groups are indicated: **P < 0.01, ***P < 0.001. (**B**) Huh7 cells were mock-treated or treated with 80 μM of RESV after DENV infection at an MOI of 1. The cells were then harvested at 24 h post infection (p.i.) and analyzed by Western blot for DENV NS3 detection. Band intensities for the ratio of DENV NS3 and β-actin were determined by Image J analysis.

To determine the effects of RESV on DENV, Huh7 cells were treated with RESV prior and post DENV infection. RESV-treated and mock-treated cells were infected with DENV at an MOI of 1. After 24 h, supernatants were harvested and the viral titers were determined using plaque assay on Vero cells. DENV-infected cells treated with RESV at different concentrations showed significant decreases of viral titers compared to untreated cells (Fig. 1A). Western blot analysis also confirmed a decrease of DENV NS3 protein production in RESV-treated cells as compared to untreated cells (Fig. 1B). We next examined whether RESV caused cytotoxicity in Huh7 cells by assaying the release of lactate dehydrogenase (LDH). The results showed no significant change in LDH release when cells were treated with RESV without DENV infection. There was a slight increase in cytotoxicity when cells were infected by DENV; however, RESV at the indicated doses did not cause a further increase in cytotoxicity (Supplementary Fig. S1). These results suggest that RESV has an antiviral activity against DENV, which is not due to any cytotoxic effect.

### RESV increases IFN-β and ISG levels during DENV infection

**Figure 2:**
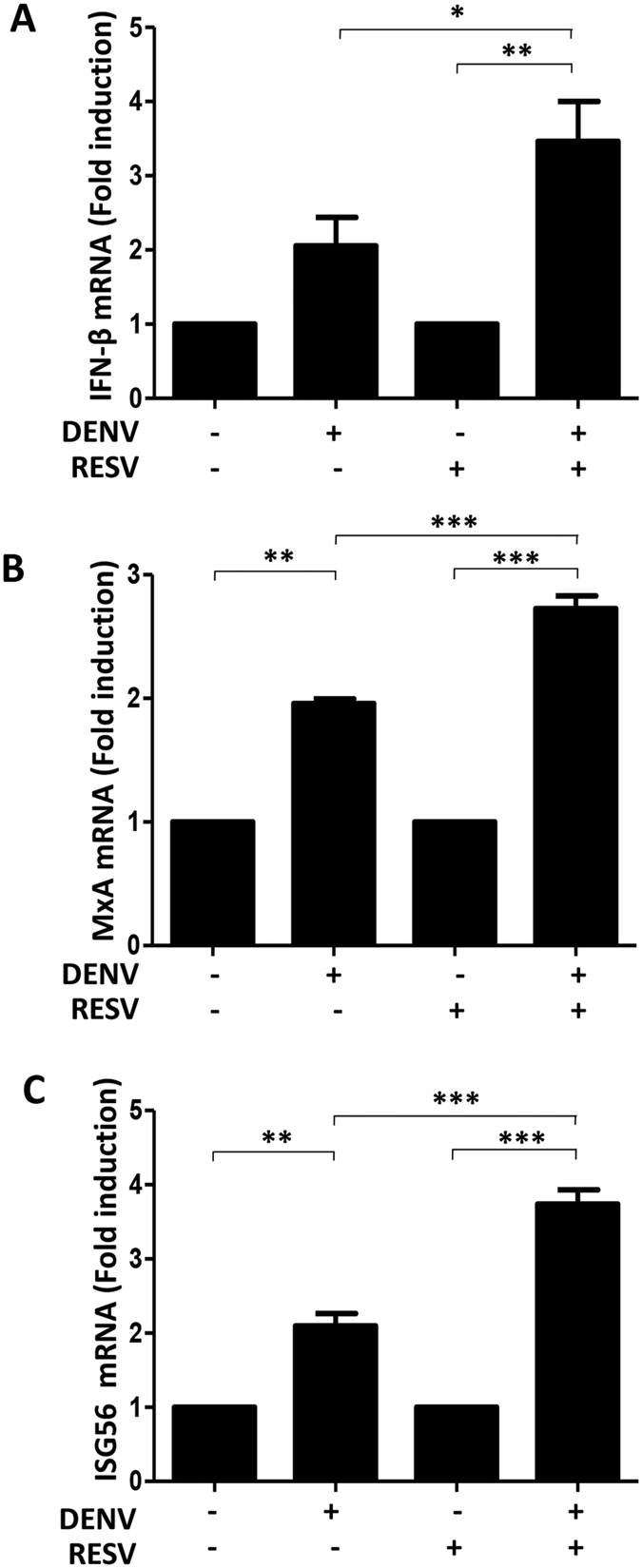
RESV increases IFN-β and ISG production in DENV-infected cells. Huh7 cells were treated with 80 μM of RESV after DENV infection at an MOI of 1. The IFN-β (**A**), MxA (**B**) and ISG56 (**C**) mRNA levels were measured by quantitative real-time RT-PCR and normalized to β-actin mRNA. Results are expressed as fold induction compared to control uninfected cells. Statistically significant differences between the groups are indicated: *P < 0.05, **P < 0.01, ***P < 0.001.

In order to investigate the mechanisms behind the antiviral activity of RESV, the type-1 IFN response was monitored by real-time quantitative PCR analysis. Compared to DENV infection alone, treatment with RESV during DENV infection showed higher level of IFN-β induction (Fig. 2A). In addition, we measured ISG mRNA levels during DENV infection with or without RESV treatment. Consistent with the increase of IFN-β, the MxA and ISG56 induction levels were significantly increased with RESV treatment as compared to without RESV treatment after DENV infection (Fig. 2B,C). Taken together, these data show that the antiviral activity of RESV against DENV may involve IFN induction and ISG activation.

### RESV inhibits translocation of HMGB1 out of nucleus during DENV infection

**Figure 3:**
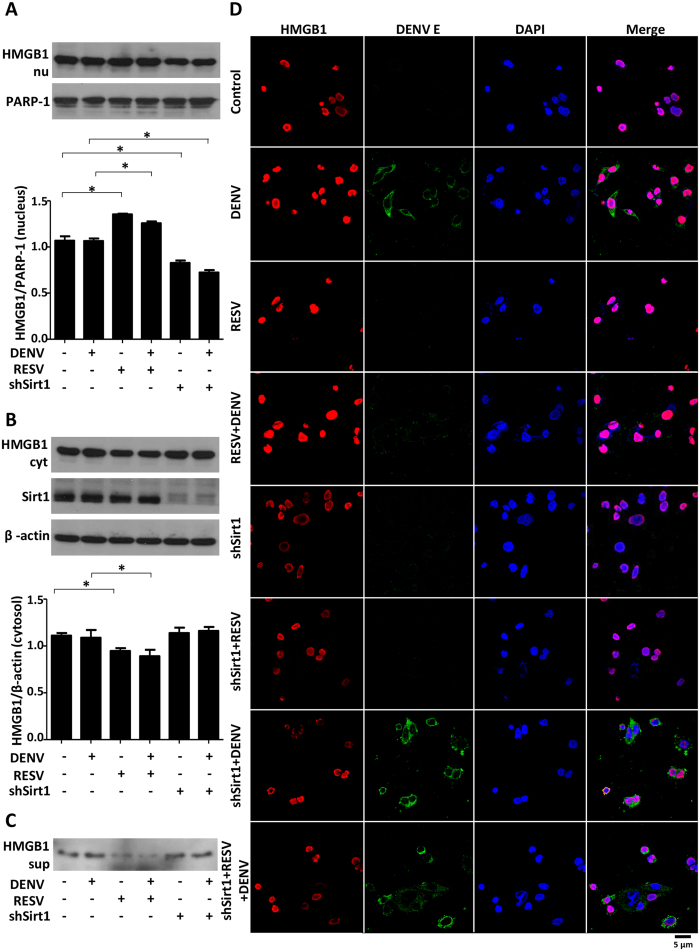
RESV increases HMGB1 in the nucleus by Sirt1-mediated translocation inhibition. Mock-treated, 80 μM RESV-treated and Sirt1-knockdown Huh7 cells were infected with DENV at an MOI of 1. Cells were harvested after 24 h p.i. and the nuclear (**A**) and cytosolic (**B**) proteins were determined by Western blot for HMGB1 detection. Statistically significant differences between the groups are indicated: *P < 0.05. (**C**) The supernatants of the cell culture were harvested and HMGB1 was measured using Western blot. (**D**) Cells were fixed after 24 h p.i. for immunofluorescence analysis (IFA). Different treatment groups are as indicated. For detection of HMGB1, cells were stained with rabbit anti-HMGB1 and donkey anti-rabbit-IgG Alexa Fluor 594 (*Red*). For detection of DENV E protein, cells were probed with mouse anti-E protein antibody and goat anti-mouse-IgG Alexa Fluor 488 (*Green*). The cell nuclei were stained with DAPI (*Blue*).

To further explore the antiviral effect of RESV, we examined the impact of the phytoalexin on HMGB1 protein. HMGB1, a DNA binding protein, usually exists in the nucleus and can be translocated out of nucleus during stress, infection or physical injury. Translocation of HMGB1 out of the nucleus in DENV-infected cell can be seen by immunofluorescence assay (IFA) (Supplementary Fig. S2A). Moreover, the detection of HMGB1 via Western blot and ELISA also showed that the HMGB1 level was higher in the supernatant from DENV-infected cells than that of mock controls (Supplementary Fig. S2B,C). RESV was found to inhibit the translocation of HMGB1 via induction of a deacetylase, Sirt1^11^. To confirm the retention of HMGB1 in the nucleus of RESV-treated cells, the cytoplasmic and nuclear proteins were assessed using Western blot analysis and immunofluorescence. RESV-treated cells showed a higher level of HMGB1 in the nucleus (Fig. 3A) and a lower level in the cytoplasm (Fig. 3B) compared to the untreated group or DENV infection alone, indicating a role for RESV in inhibition of HMGB1 translocation out of the nucleus. Moreover, Sirt1-knockdown cells demonstrated a prominent reduction of HMGB1 in the nucleus, confirming the effect of Sirt1 in inhibiting HMGB1 translocation (Fig. 3A). Similar to the pattern of HMGB1 level in the cytosol, the amount of HMGB1 in the supernatant of RESV-treated cells was reduced compared to the untreated group or DENV infection alone due to the retention of HMGB1 in the nucleus, whereas Sirt1-knockdown cells showed higher amount of HMGB1 in the supernatant due to the increased translocation of HMGB1 out of the nucleus (Fig. 3C). Using IFA we further determined the intracellular location of HMGB1 and observed a higher intensity of HMGB1 after RESV treatment. RESV caused accumulation of HMGB1 in the nucleus of DENV-infected cells and resulted in an inhibition of DENV as shown by DENV E detection (Fig. 3D, panel 4 as compared with panel 2). Moreover, Sirt1-knockdown cells displayed a lower intensity of HMGB1 in the nucleus, confirming the inhibition of HMGB1 translocation by RESV treatment. Treatment with RESV in Sirt1-knockdown cells neither increased HMGB1 accumulation in the nucleus nor inhibited DENV replication (Fig. 3D).

### HMGB1 localized in the nucleus is required for the antiviral response of RESV

**Figure 4:**
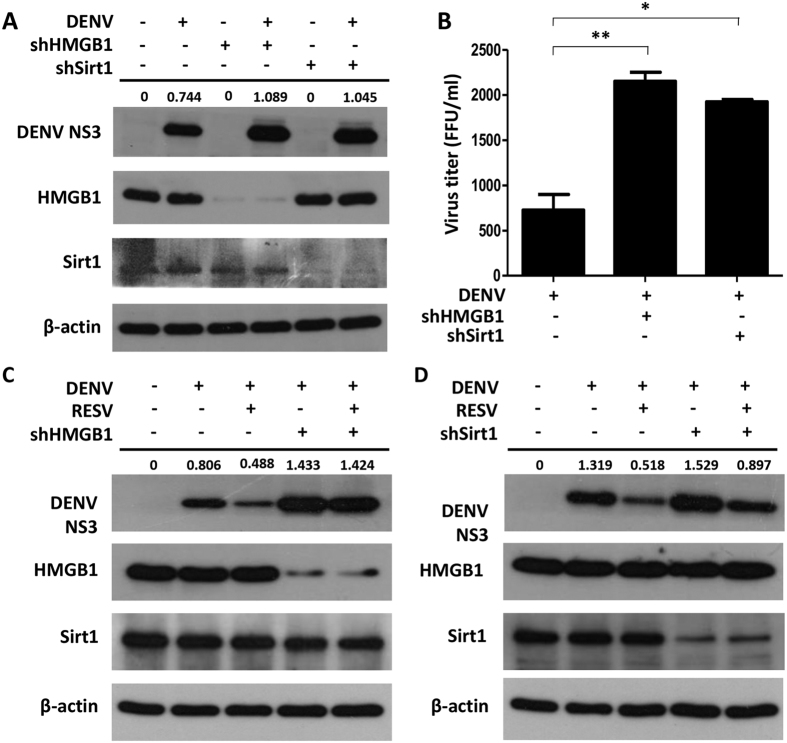
Antiviral activity of RESV relies on the presence of HMGB1 and Sirt1. (**A**) Huh7 WT, shHMGB1 and shSirt1 cells were infected with DENV at an MOI of 1. Cells were harvested at 24 h p.i. and DENV NS3, HMGB1 and Sirt1 were detected by Western blot analysis. (**B**) The supernatants of the cell culture were harvested and virus titers were detected using focus assay. Statistically significant differences between the groups are indicated: *P < 0.05, **P < 0.01. (**C**,**D**) Huh7 WT, shHMGB1 and shSirt1 cells were mock-treated or treated with 80 μM RESV after infection with DENV. Levels of DENV NS3, HMGB1 and Sirt1 were analyzed using Western blot. Band intensities for the ratio of DENV NS3 and β-actin were determined by Image J analysis.

We then determined the role of HMGB1 in the antiviral response mediated by RESV by assessing the DENV replication in HMGB1-depleted cells. Our results showed that DENV replication as indicated by NS3 production and virus titers were higher in the HMGB1-knockdown cells as compared to the DENV infection alone control cells, suggesting that HMGB1 is required in the host antiviral response (Fig. 4A,B). Knockdown of Sirt1, a protein that inhibits HMGB1 translocation, also resulted in higher DENV replication than DENV infection alone control cells (Fig. 4A,B). These results suggested that the lack of HMGB1 in the cells (HMGB1-knockdown) or reduced amount of HMGB1 in the nucleus (Sirt1-knockdown) leads to a less efficient antiviral response.

Next, we validated that the antiviral activity of RESV treatment relied on the presence of HMGB1 in the nucleus. In order to achieve this, the effect of RESV treatment in HMGB1-depleted cells was determined. DENV-infected HMGB1-knockdown cells showed no change of NS3 production while wild-type (WT) cells showed a decrease after RESV treatment (Fig. 4C), signifying that the absence of HMGB1 in the cells disrupts the antiviral activity of RESV. Furthermore, Sirt1 knockdown which causes decreased HMGB1 in the nucleus also showed less reduction of NS3 production than WT cells with RESV treatment (Fig. 4D). These results imply a novel role for RESV in regulating nuclear HMGB1 against DENV infection.

### HMGB1 in the nucleus plays a role in the induction of ISGs

**Figure 5:**
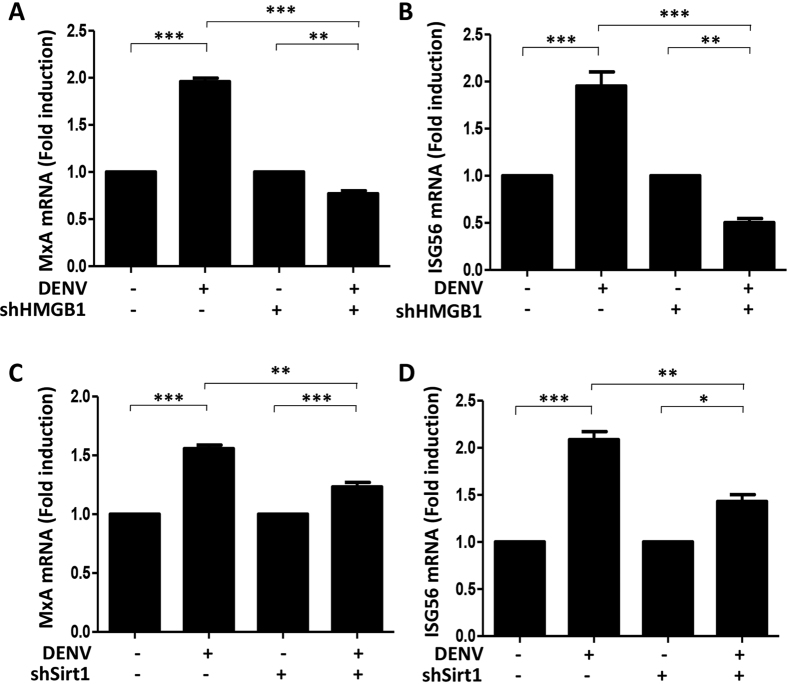
HMGB1 and Sirt1 are required to trigger ISG expression in DENV-infected cells. (**A**,**B**) Huh7 WT and shHMGB1 cells were mock-infected or infected with DENV at an MOI of 1. Cells were harvested at 24 h p.i. and MxA (**A**) and ISG56 (**B**) mRNA were quantified using quantitative real-time RT-PCR, normalized to β-actin mRNA level. (**C**,**D**) Huh7 WT and shSirt1 cells were mock-infected or infected with DENV at an MOI of 1. Cells were harvested at 24 h p.i. and MxA (**C**) and ISG56 (**D**) mRNA were detected using RT-PCR. Statistically significant differences between the groups are indicated: *P < 0.05, **P < 0.01, ***P < 0.001.

Given our findings on the less efficient RESV-mediated antiviral response in HMGB1 and Sirt1-knockdown cells, we further determined the effect of HMGB1 on ISG induction. Real-time quantitative PCR analysis showed that the mRNA expression of ISGs (MxA and ISG56) in both HMGB1- and Sirt1-knockdown cells were significantly reduced compared to WT cells (Fig. 5), complementing the findings in Fig. 2 that HMGB1 restricted in the nucleus by RESV resulted in the increase of ISG levels.

### Nuclear HMGB1 mediates IFN-β-induced ISG activation

**Figure 6:**
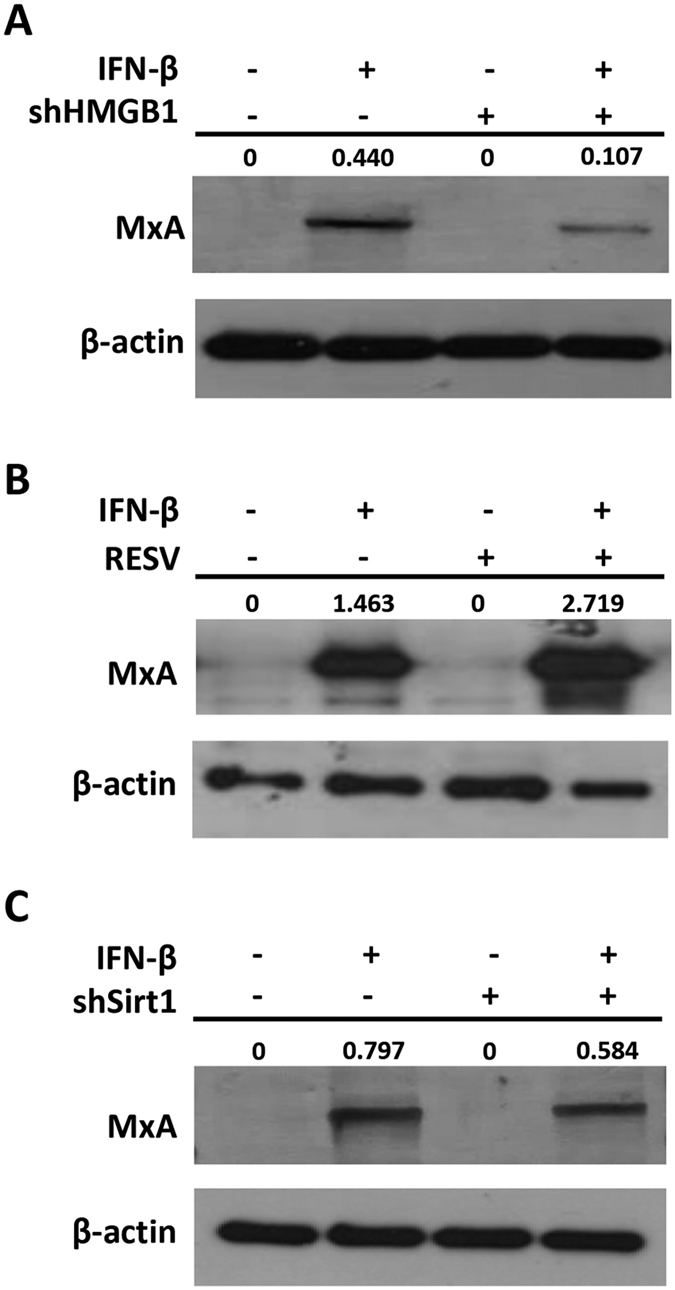
HMGB1 and Sirt1 regulate the induction of MxA in response to IFN-β. HMGB1-knockdown cells (**A**, shHMGB1) and Sirt1-knockdown cells (**C**, shSirt1) were stimulated with 50 μM IFN-β for 16 h and the MxA levels were determined using Western blot analysis. Huh7 cells were treated with 80 μM RESV (**B**, RESV-treated) for 4 h before and during the stimulation of 50 μM IFN-β for 16 h and the MxA levels were determined using Western blot analysis. Band intensities for the ratio of MxA and β-actin were determined by Image J analysis.

In order to determine the involvement of HMGB1 in the pathway leading to the activation of ISGs, MxA expression was measured after IFN-β stimulation in the HMGB1-knockdown (Fig. 6A), RESV-treated (Fig. 6B), and Sirt1-knockdown cells (Fig. 6C). Western blot analysis showed that the MxA level in HMGB1-knockdown cells was decreased, suggesting the importance of HMGB1 in ISG expression. RESV-treated cells displayed an increase in MxA level, indicating that the accumulation of HMGB1 in the nucleus causes upregulation of ISG expression in response to IFN-β. Moreover, Sirt1-depleted cells which, like HMGB1-knockdown cells, lack nuclear HMGB1, also showed decreased MxA expression in response to IFN-β, further indicating that nuclear-localized HMGB1 contributes to the regulation the antiviral response.

### HMGB1 binds to the promoter region of MxA

**Figure 7:**
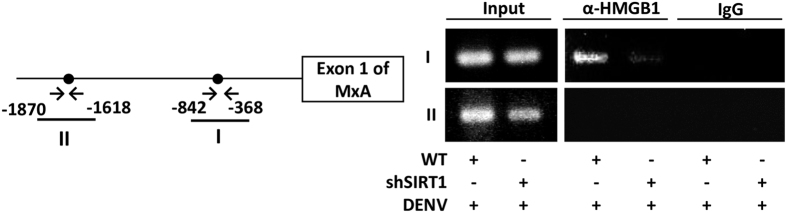
HMGB1 binds to the promoter region of MxA. Primers were used to amplify MxA promoter fragment encompassing the proximal HMGB1 binding site (I: from −842 to −368) or distant from the HMGB1 binding site as a negative control (II: from −1870 to −1618). The chromatin immunoprecipitation assay was performed with the immunoprecipitation products identified by the indicated antibodies from WT or Sirt1-knockdown Huh7 cells infected with DENV at an MOI of 1 for 12 h. Primers I and II were used to amplify the MxA promoter fragment.

Next, we determined whether HMGB1 could act as a transcriptional regulator of the MxA gene. To explore whether HMGB1 may bind to the promoter region in *MxA* during DENV infection, we performed an *in vivo* DNA binding assay using chromatin immunoprecipitation. The results showed that HMGB1 binds to the region between −842 and −368 from the start codon after DENV infection, and the binding was decreased in Sirt1-depleted cells (Fig. 7). We also confirmed that HMGB1 binds to the promoter region of MxA after DENV infection, but not in the group without DENV infection (Supplementary Fig. S3). The results suggest that DENV infection triggers HMGB1 binding to the promoter region of MxA and Sirt1 knockdown reduces the level of HMGB1 in the nucleus. This then results in diminished DNA-protein complexes as shown by the reduced binding of HMGB1 with the promoter of MxA.

## Discussion

**Figure 8:**
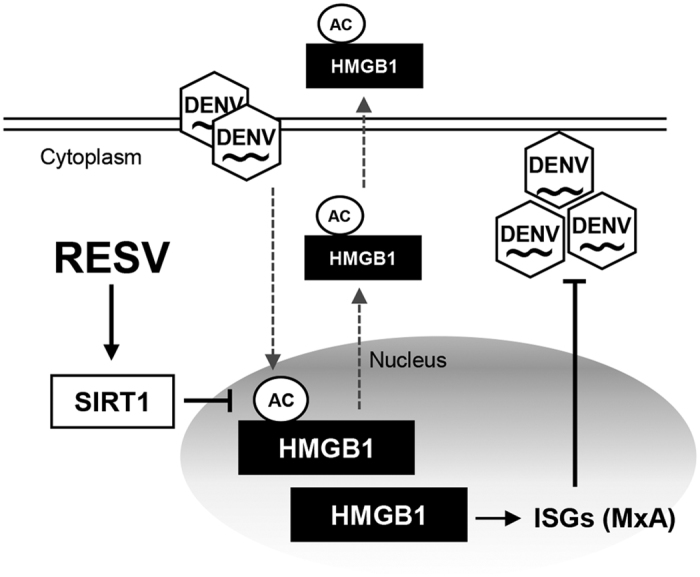
Model of anti-DENV cell response by RESV treatment. DENV infection triggers the acetylation of HMGB1 in the nucleus, causing the release of HMGB1 into the cytoplasm and extracellular milieu. RESV treatment induces production of Sirt1, leading to deacetylation of HMGB1. This then reduces the translocation of HMGB1 and increases the accumulation of HMGB1 in the nucleus. The higher amount of nuclear HMGB1 enhances the production of ISGs, leading to efficient antiviral response in the cell.

The results presented here indicate a previously unknown role for HMGB1 in the regulation of ISGs and its antiviral response. The inhibitory effect of RESV on DENV is mediated through the regulation of ISGs, which in turn are regulated by HMGB1 accumulation in the nucleus. These findings are summarized schematically in Fig. 8.

RESV has been reported to possess antiviral activity against a number of different viruses^7^. However, the exact mechanism of the viral inhibition is still not fully defined. Several mechanisms have been suggested, including blocking of NF-κB pathways, inhibition of nucleotide reductase action, blocking of EGR-1, interference with autophagy pathways, and manipulation of sirtuins^27^. In our study, we showed that the inhibition of DENV replication by RESV treatment was likely due to a higher expression of ISGs that leads to enhanced antiviral response. MxA, one of the ISGs, exerts antiviral activity in the early steps of virus replication^28^. It was previously shown that both dimeric and monomeric variants of MxA are capable of forming complexes with nucleoprotein of influenza A virus, thus blocking virus replication^29^. Moreover, mutant mice carrying human MxA were protected from fatal influenza A virus infection^29^. Another ISG protein, ISG56, inhibits HCV replication by repressing the internal ribosome entry site (IRES)-mediated transcription of the virus^30^. Numerous studies have illustrated the importance of ISGs in the antiviral response^31^.

An interesting finding in our study was the role of HMGB1 as a mediator of the regulation of ISGs following RESV treatment. HMGB1 is considered a biomarker or a therapeutic target of certain diseases. HMGB1 is usually detected at the later stages of infection when it is translocated out of the nucleus and further secreted out of the cell^16^. However, the potential regulatory roles of HMGB1 in the nucleus are less understood. We demonstrated that RESV inhibits translocation of HMGB1 which leads to the accumulation of HMGB1 in the nucleus. Inhibition of HMGB1 translocation out of the nucleus by RESV treatment is shown to be mediated by Sirt1. Even though the earlier descriptions of antiviral effects of RESV have been associated with sirtuins, the possible mechanisms by which RESV inhibits virus infection and pathogenesis require further investigation^32^. A previous study suggested that RESV induces Sirt1 that leads to deacetylation of HMGB1, restricting the translocation of HMGB1 out of the nucleus^11^. Our data showed that the inhibition of HMGB1 translocation during DENV infection by RESV treatment increases the level of ISGs, leading to an enhanced antiviral response. Using the ChIP assay, we demonstrated that HMGB1 binds to the promoter region of an ISG, MxA. In addition, Sirt1-knockdown cells displayed lower binding activity between HMGB1 and the promoter region of MxA, which is associated with the reduced HMGB1 in the nucleus of the cell. Therefore, we deduced that HMGB1 transcriptionally promotes the expression of MxA. The accumulation of HMGB1 in the nucleus by RESV treatment thus promotes the expression of ISGs, such as MxA, thereby enhancing antiviral response. It should be noted that our results do not provide evidence for direct binding of HMGB1 with DNA, it cannot be excluded that HMGB1 may bind to another protein such as a transcription factor which binds DNA.

There is ongoing extensive research on the prevention of dengue disease, including antiviral development, vaccine production, and management of mosquitoes. Despite the recent approval of a DENV vaccine in several countries, weak efficacy remains a concern^33^. In addition, pronounced hyper-activity of immune responses of host against the virus may also contribute to severe dengue disease. Thus, the search for antivirals and other effective treatment measures for DENV infection remain crucial.

Recent work suggested that extracellular HMGB1 contributes to vascular endothelial leakage during DENV infection^19^. DENV-induced translocation of HMGB1 from the nucleus to cytoplasm is triggered by HMGB1 acetylation that leads to its release. The released HMGB1 may bind to RAGE, TLR2 or TLR4 and then activates NF-κB which upregulates pro-inflammatory genes, which may be associated with the disease pathogenesis of DHF/DSS^15^,^19^. RESV may possess properties useful as an antiviral against dengue. In addition to its ability to antagonize DENV replication, it may also reduce induction of vascular leakage and activation of pro-inflammatory genes in dengue. In addition, extracellular HMGB1 was also found to induce macrophage inflammatory responses in systemic lupus erythematosus^34^. Hence, reduction of HMGB1 release by treatment with RESV may decrease the amount of extracellular HMGB1, thus potentially reducing induction of vascular leakage and activation of pro-inflammatory genes. In addition, RESV also suppresses the NF-κB and JAK/STAT signaling pathways via the inhibition of HMGB1 release out of the cell^20^.

In conclusion, RESV partially suppresses DENV replication by increasing the activation of ISGs through inhibition of HMGB1 translocation. The inhibitory effect of RESV on HMGB1 translocation out of the nucleus also suggests the possibility that RESV treatment may downregulate pro-inflammatory genes during DENV infection. This study provides insights into the possible mechanisms of action of RESV and introduces a novel role for HMGB1 in the host antiviral response against DENV.

## Materials and Methods

### Ethics statement

Human serum used in this research was obtained from dengue positive individuals with informed written consent. The procedure of sample collection was approved (National Cheng Kung University Hospital No. A-BR-101-140) and carried out in accordance with the Institutional Review Board of National Cheng Kung University Hospital, which is organized and operated according to the laws and regulations of Good Clinical Practice (ICH-GCP).

### Cell and virus

Human hepatocellular carcinoma cells (Huh7) were maintained in Dulbecco’s modified Eagle medium (DMEM) (Life Technologies, Carlsbad, CA) supplemented with 1% penicillin/streptomycin and 10% heat-inactivated fetal bovine serum (FBS) (Biological Industries, Kibbutz Bet Haemek, Israel). Huh7 cells were incubated at 37 °C in a 5% CO_2_, humidified incubator. Huh7 cell line was purchased from the American Type Culture Collection (ATCC; Manassas, VA). Dengue virus (DENV) serotype 2 strain PL046 used in this study was propagated in C6/36 mosquito cells and plaque or chromogenic focus assay were carried out to quantify virus titers.

### Stable knockdown cells

Lentiviruses expressing shRNAs to knockdown HMGB1 and Sirt1 were prepared by the National RNAi Core Facility, Academia Sinica, Taiwan. Huh7 cells were infected with the lentiviruses at a multiplicity of infection (MOI) of 3 in the presence of polybrene (hexadimethrine bromide; Sigma-Aldrich Chemical, St. Louis, MO) at a final concentration of 8 μg/ml. Cells were incubated for 24 h, and then the medium was replaced with selective medium containing puromycin (Sigma-Aldrich Chemical) at a final concentration of 3 μg/ml.

### Viral infection and evaluation of viral titers

Huh7 cells were infected with DENV with or without resveratrol (RESV) (Sigma-Aldrich Chemical) added to cells at various concentrations. Viral titers in culture supernatants were determined as plaque- or focus-forming units (PFU or FFU)/ml. For this, 1 × 10^5^ BHK or Vero cells per well in 12 or 24-well plates (Costar, Corning, NY) were inoculated with serially diluted supernatants (lowest dilution 1:2). The plaque assay was performed and stained with crystal violet (Sigma-Aldrich Chemical). For FFU assay, after infection for 72 h, cells were fixed with 4% paraformaldehyde in phosphate-buffered saline (PBS) for 20 min and permeabilized with 1% Triton X-100 in PBS for 15 min at room temperature (RT). After three washes with PBS, fixed cells were blocked with 3% skimmed milk in PBS for 2 h at RT. Cells were then stained with primary antibody (1:500 of human serum) and secondary antibody (1:250 of peroxidase-conjugated goat anti-human-IgG; Jackson ImmunoResearch Laboratories, West Grove, PA) for 1 h at 37 °C. The immunostained cells were visualized with a 3, 3′-diaminobenzidine (DAB) substrate (Vector Laboratories, Burlingame, CA). FFUs were counted and infectivity titers were calculated.

### Western blot analysis

Huh7 cell cultures and supernatants were harvested at 24 h post infection. Proteins were obtained from the Huh7 cells using a cytoplasm and nuclear protein extraction kit (ThermoScientific, Waltham, MA) or total protein extraction reagent. For total protein extraction, cells were washed, scraped and subjected to lysis buffer (10 mM Tris-HCl, 50 mM NaCl, 5 mM EDTA, 10 mM NaN_3,_ 10 mM NaF, 10 mM Na_4_O_7_P_2_, 1% Triton X-100, pH 7.5). Cell extracts and supernatants were mixed with 25% of sample buffer (250 mM Tris-HCl, 500 mM DTT, 10% SDS, 0.1% bromophenol blue, 50% glycerol). Protein concentrations were determined using the Bradford Assay, and equal amounts of protein were loaded in individual wells and resolved by SDS-PAGE. Proteins were then transferred onto nitrocellulose membranes using HEP-1 Panther semi-dry electroblotter system (ThermoScientific, Waltham, MA). Membranes were blocked with 5% skimmed milk in PBS and subsequently probed overnight with primary antibodies at 4 °C. The primary antibodies were rabbit anti-HMGB1 antibody (1:2500 dilution; Abcam, Cambridge, UK), rabbit anti-NS3 antibody (1:5000 dilution; GeneTex, San Antonio, TX), rabbit anti-MxA (1:1000 dilution; Abcam) and rabbit anti-ISG15 (1:1000 dilution; Cell Signaling Technology, Danvers, MA). Blots were then incubated with secondary antibody, horseradish peroxidase (HRP)-conjugated anti-rabbit IgG (1:5000 dilution; Cell Signaling Technology) for 1 h at RT. Proteins were detected using the Immobilon Western Chemiluminescent HRP substrate kit (Millipore, Saint Louis, MO) and exposed to film.

### Quantitative real-time PCR

Total RNA was extracted from Huh7 cells using Direct-Zol miniprep kit (Zymo Research, Irvine, CA). Reverse transcription of RNA was performed using PrimeScript RT reagent kit (TaKaRa Bio. Inc., Shiga, Japan). Quantitative real-time PCR was carried out using SYBR Green PCR master mix (Applied Biosystems, CA). PCR primers used in this study are listed in Supplementary Table S1. The mRNA levels were shown as fold induction compared to uninfected cells and normalized to intracellular β-actin mRNA levels.

### Immunofluorescence microscopy

Huh7 cells were infected with DENV with or without RESV. After 24 h of infection, cells were harvested and fixed using 4% paraformaldehyde in PBS for 20 min at RT. Cells were double stained with primary antibodies including rabbit anti-HMGB1 antibody (1:1000 dilution; Abcam) and mouse anti-E antibody (1:500 dilution; GeneTex, San Antonio, TX) and kept overnight at 4 °C. Cells were then incubated with secondary antibodies including donkey anti-rabbit-IgG Alexa Fluor 594 and goat anti-mouse-IgG Alexa Fluor 488 (Life Technologies) at a dilution of 1:250 for 1 h at RT. For counterstaining of the nuclei, cells were incubated with 4′ 6′-diamino-2-phenylindole dihydrochloride (DAPI) at a dilution of 1:1000 for 15 min before washing with PBS. The samples were mounted onto glass slides for confocal microscopy (Olympus, FV1200) or a BX51 Olympus fluorescence microscope (Olympus, Melville, NY).

### Chromatin immunoprecipitation assay

Huh7 cells were treated with 1% formaldehyde for 15 min. The cross-linked chromatin was then prepared and sonicated to an average size of 500 bp. The DNA fragments were immunoprecipitated with specific antibodies recognizing HMGB1 or control rabbit IgG at 4 °C for 12–16 h. After reversal of the crosslinking between proteins and genomic DNA, the precipitated DNA was amplified by PCR with primers related to the specific regions on the genomic loci of target genes. The primers for MxA-I and MxA-II are listed in Supplementary Table S1.

### Statistical analysis

Experimental groups were compared using a one-way analysis of variance (ANOVA) followed by Tukey’s test to show if the variances differed significantly, followed by Bonferroni’s multiple-comparison test. The results were considered significantly different when *P* was < 0.05.

## Additional Information

**How to cite this article**: Zainal, N. *et al*. Resveratrol treatment reveals a novel role for HMGB1 in regulation of the type 1 interferon response in dengue virus infection. *Sci. Rep.* **7**, 42998; doi: 10.1038/srep42998 (2017).

**Publisher's note:** Springer Nature remains neutral with regard to jurisdictional claims in published maps and institutional affiliations.

## Supplementary Material

Supplementary Information
