# Supplementary material for: Resveratrol treatment reveals a novel role for HMGB1 in regulation of the type 1 interferon response in dengue virus infection
Source: Sci Rep. 2017 Feb 20;7:42998. doi: 10.1038/srep42998 (PMC5316936; doi:10.1038/srep42998)
Supplement: Supplementary Information [file srep42998-s1.pdf]

Supplementary Information

Resveratrol treatment reveals a novel role for HMGB1 in regulation of the type 1 interferon response in dengue virus infection

Nurhafiza Zainal, Chih-Peng Chang, Yi-Lin Cheng, Yan-Wei Wu, Robert Anderson, Shu-Wen Wan, Chia-Ling Chen, Tzong-Shiann Ho, Sazaly AbuBakar, and Yee-Shin Lin*

Supplementary Table 1. List of primer sequences used in this study.

Primer	Forward	Reverse
IFN-β	TAGCACTGGCTGGAATGAGA	TCCTTGGCCTTCAGGTAATG
ISG56	GGGCA GACTG GCAGA AGC	TATAG CGGAA GGGAT TTGAA AGC
MxA	ACCAC AGAGG CTCTC AGCAT	CTCAG CTGGT CCTGG ATCTC
β-actin	AAGGA GAAGC TGTGC TACGTCGC	AGACA GCACT GTGTT GGCGT ACA
MxA-I	GCAGC CATCT CAAAG TATGC	AGGAG CAGAA GCTGA AATCC
MxA-II	TGGAG AGGAA CAGCA GAGG	GCATT CAGCA CATGA TCG

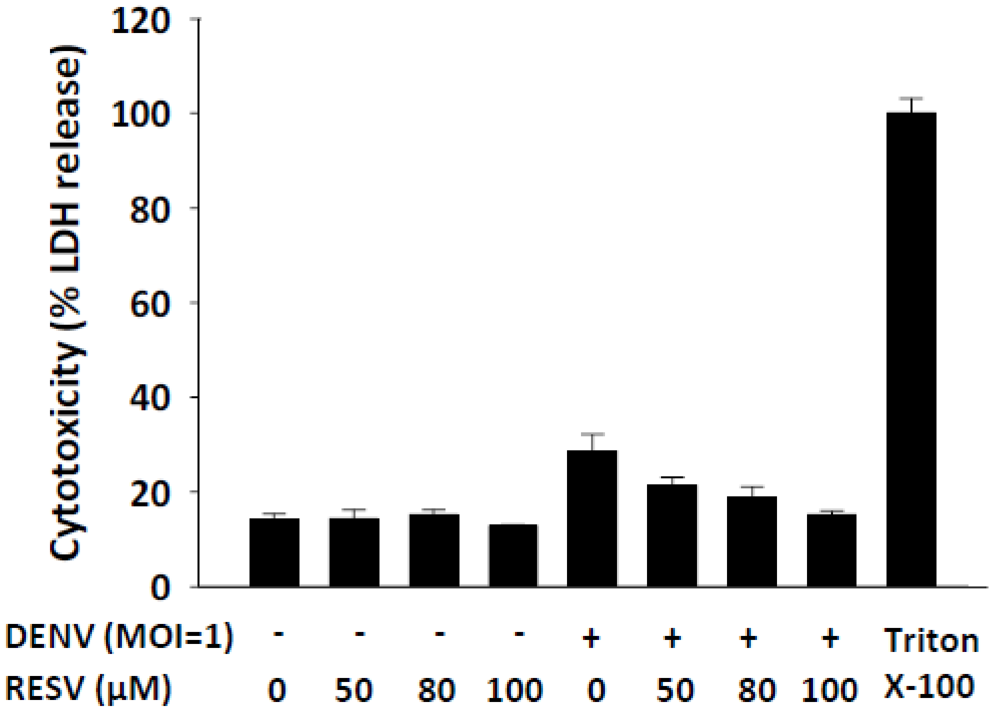

Supplementary Figure 1. RESV treatment does not cause cytotoxicity in Huh7 cells. Huh7 cells were mock-treated or treated with 50, 80 or 100 µM of RESV after DENV infection at an MOI of 1. After 24 h, cell culture supernatants were collected and assayed for the release of lactate dehydrogenase (LDH). Triton X-100 served as a positive control.

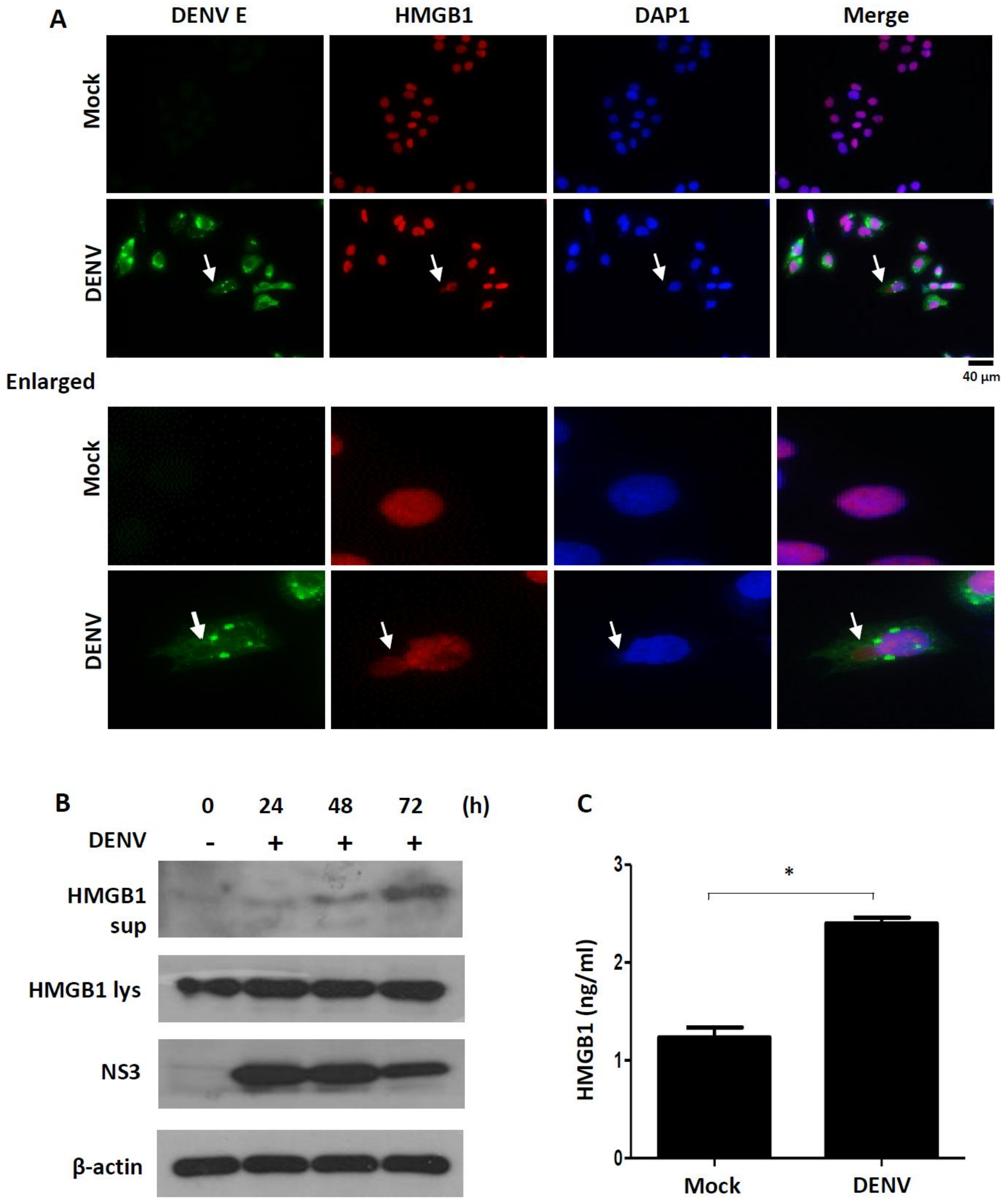

Supplementary Figure 2. DENV infection induces translocation of HMGB1 from the nucleus to the extracellular medium. (**A**) Huh7 cells were infected with DENV at an MOI of 10. After 24 h, cells were fixed for immunofluorescence analysis (IFA). For detection of HMGB1, cells were stained with rabbit anti-HMGB1 and donkey anti-rabbit-IgG Alexa Fluor 594 (red). For detection of DENV E protein, cells were stained with mouse anti-E protein antibody and goat anti-mouse-IgG Alexa Fluor 488 (green). Cell nuclei were stained with DAPI (blue). Arrows indicate cytosolic HMGB1. (**B**) Cell lysates and the supernatants were collected at 24 h p.i. and analyzed by Western blot for DENV NS3 and HMGB1 detection.

(**C**) HMGB1 levels were analyzed by ELISA. Statistically significant differences between the groups are indicated: *, $P < 0.05$.

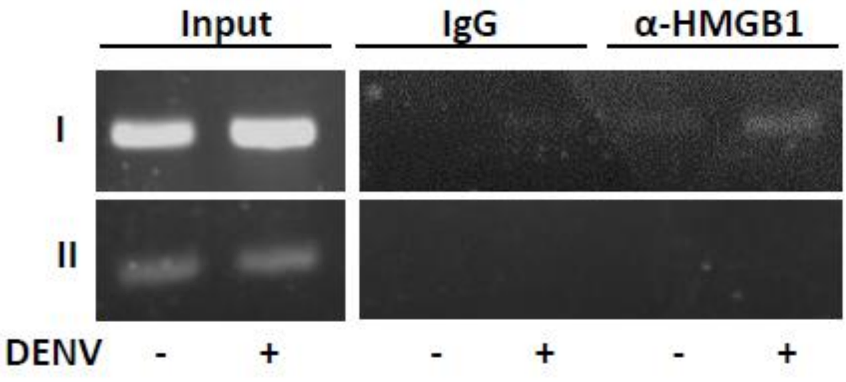

Supplementary Figure 3. HMGB1 binds to the promoter region of MxA after DENV infection. Primers were used to amplify the MxA promoter fragment encompassing the proximal HMGB1 binding site (I: from -842 to -368) or distant from the HMGB1 binding site as a negative control (II: from -1870 to -1618). The chromatin immunoprecipitation assay was performed with the immunoprecipitation products identified by the indicated antibodies from Huh7 cells noninfected or infected with DENV at an MOI of 1 for 12 h. Primers I and II were used to amplify the MxA promoter fragment.
